# Developing Criteria for Lumbar Spine Magnetic Resonance Imaging (MRI) Using RAND Appropriateness Method (RAM)

**DOI:** 10.5812/iranjradiol.4063

**Published:** 2012-09-17

**Authors:** Ali Keshtkaran, Mohammad Hadi Bagheri, Rahim Ostovar, Hedayat Salari, Majid Reza Farokhi, Atefeh Esfandiari, Hossein Yousefimanesh

**Affiliations:** 1 Department of Medical Informatics and Management, Shiraz University of Medical Sciences, Shiraz, Iran; 2 Department of Radiology, Shiraz University of Medical Sciences, Shiraz, Iran; 3 Social Determinants of Health Center Research, Yasuj University of Medical Sciences, Yasuj, Iran; 4 Department of Neurosurgery, Shiraz University of Medical Sciences, Shiraz, Iran; 5 Department of Health Services Administration, Kerman Medical Students Research Committee, Kerman University of Medical Sciences, Kerman, Iran; 6 Department of Medicine, Shiraz Medical Students Research Committee, Shiraz University of Medical Sciences, Shiraz, Iran

**Keywords:** Lumbar Vertebrae, Magnetic Resonance Imaging, RAND, RAM, Clinical Scenario

## Abstract

**Background:**

Studies show that a large proportion of healthcare offered may be inappropriate or unnecessary. Magnetic resonance imaging (MRI) is a new and expensive diagnostic technology which has been increasingly used all over the world. Moreover, this trend has been more rapidly increasing in Iran. Low back pain is a common disorder all over the world and MRI technique is one of the several ways to assess its cause.

**Objectives:**

The present study aims to develop scenarios for lumbar spine MRI.

**Materials and Methods:**

In the present study, the RAND Appropriateness Method (RAM) was used in order to reach consensus regarding developing scenarios for lumbar spine MRI. We generated scenarios from valid clinical guidelines as well as the experts’ opinion. The panel members included nine specialists from various medical specialties that had scored scenarios in two rounds, the first of which was without interaction, while the second one was with interaction.

**Results:**

We extracted 97 scenarios for the lumbar spine MRI in the scenario extracting phase of the study and the panel members added 18 scenarios. After implementation of two rounds, the scenarios were categorized into three ranges. Sixty seven (58%) of the scenarios were considered as appropriate, 45 (39%) as uncertain, and three (2.6%) as inappropriate.

**Conclusions:**

RAM is useful for identifying stakeholder views in settings with limited resources. Since RAM has precise instructions for consensus developing, a large number of scenarios were considered as uncertain. Therefore, more research has to be conducted on the issue.

## 1. Background

Healthcare systems face significant and rapid changes in response to changes in population needs and increasing costs (1, 2). Studies show that a large proportion of the healthcare offered is inappropriate or unnecessary, ranging from 15 to 30 percent in many countries and as much as 40 percent in some private clinics (3, 4). The quality of and access to healthcare are not related to high expenditures in the health system (5). Medical imaging costs have had an incremental trend worldwide which might be due to overuse. In fact, among all medical imaging technologies, magnetic resonance imaging (MRI) usage has noticeably had a more incremental trend (6, 7, 8, 9, 10). In case physicians order inappropriately demanded medical imaging procedures, it may result in serious problems in terms of both healthcare economy and quality (11). Moreover, medical imaging overuse might be unable to improve the patients’ health status. As studies have shown, 20% - 50% of high-tech imaging procedures have been unnecessary. (12, 13, 14, 15). Various methods have been developed in order to measure the appropriateness of health care and to develop valid criteria for the physicians (1, 2, 16, 17). Financial and human resources are needed in order to develop the clinical guidelines and these resources may not be available for low and middle income countries (18). Due to these limitations, we aimed to identify the criteria for using available valid guidelines.

## 2. Objectives

Since low back pain (LBP) is one of the most prevalent disorders (19, 20) and there is little consensus among the specialists about appropriate LBP procedures (21, 22), we aimed to identify the appropriate criteria for utilization of MRI in LBP patients based on the viewpoints of the experts in the country. The result of this study may be useful for policymakers as well as clinicians in Iran and in other low and middle income countries.

## 3. Patients and Methods

RAND Appropriateness Method (RAM) was selected for the present study since it allows the development of the appropriateness criteria based on the available evidence which is supplemented by the expert panels’ opinion. This method was designed in the 1980s by the RAND institute and the University of California in Los Angeles (UCLA) and has been used in many studies in North America and Europe. RAM involves generating clinical scenarios or criteria. RAM has been used in many studies (2, 23) especially for development of the appropriateness criteria in surgical care as well as investigative procedures (2, 24, 25, 26). The present study was conducted in Shiraz in 2011. Besides, followed is a modified version of RAM which is explained in the next section.

### 3.1. Generating Scenarios

We started the study by searching for available evidence. In the process of the literature review, we focused on clinical practice guidelines and evidence reviews. In this way,10 relevant documents were identified (27, 28, 29, 30, 31, 32, 33, 34, 35). Then, we reviewed and verified the identified documents and selected four guidelines and evidence reviews which provided a relatively comprehensive coverage of the issues related to MRI for LBP (34, 35). Two panel members as well as two authors used the Persian translation of the AGREE (Appraisal of Guidelines for Research and Evaluation) tool (36) in order to assess the guidelines and finally selected two of them from which the indications and the scenarios were extracted. Each indication was divided into several scenarios and each scenario included a few words which described the patients’ signs. First, we identified the major specialists who were involved in the prescription of MRI for low back pain. we found that six majors were involved in this issue. Then, nine specialists were invited to participate in the panel.

### 3.2. Panel Members

The panel consisted of nine specialists: two neurosurgeons, two orthopedic surgeons, two radiologists, one neurologist, one rheumatologist and one physiatrist, all of whom were faculty members and worked in educational hospitals. Before the process of consensus making, we talked to all the panelists and described the objectives of the study as well as the process of consensus making. If they agreed, we invited them to participate in the study.

### 3.3. Consensus Making

The process of consensus making among the specialists was implemented in two rounds: without interaction and with interaction. In the first round, we sent the first form (including the list of scenarios, summaries of the clinical guidelines and the scoring system) to each expert panelist. We asked them to select the best score for each scenario based on their professional judgment and summary of evidence. The scores ranged from one (absolutely inappropriate) to nine (absolutely appropriate). Then, they sent the scored scenarios back to us. We entered all the specialists’ scores to the second form and prepared it for the second round of consensus development. The second forms provided an opportunity for each panelist to be informed about the other panelists’ scores anonymously. In the second round, the members were invited to a meeting. In the meeting, we gave the second form to all the panel members. One author (MHB) was selected as the panel leader. He read each scenario, asked the panelists about each scenario and if necessary, changed their first round scores. The meeting lasted for six hours. Then, the new indications presented by the specialists were collected and discussed. In the meeting, it was emphasized that the panelists should score the scenarios independently while considering the panel discussions.

### 3.4. Statistical Analysis

Median scores were used to judge the appropriateness of the scenarios. The scores were categorized into three groups: appropriate (score = 7-9), uncertain (score = 4-6), and inappropriate (score = 1-3). Of course, in order to reach agreement, another condition had to be met: if minimum and maximum scores are ignored, all the other scores must fall in the same scoring group. 

## 4. Results

We extracted 97 scenarios for the lumbar spine MRI in the scenario extracting phase of the study. We divided the scenarios into sixteen indications, as used in the selected guidelines (34, 35), in order to make the assessment easier for the panel members (23). Totally, over eighty nine percent of the scenarios were considered as appropriate, but the agreement was reached on 72 (74%) of them. Only three scenarios were considered inappropriate at the first round. According to the results, the second and the fifth indications had the highest extracted scenarios. Comparing the indications, the tenth indication contained the highest number of appropriate scenarios with agreement (Table 1). In the second round of consensus making, the panel members added 18 indications to the first list, all of which were appropriate, but the agreement on them was zero. After discussion during the second panel, four indications were ignored from the list and the consensus process was done on 30 indications. The rate of appropriateness and agreement of scenarios in the second round is presented in Table 2. It is seen that among the 111 scenarios of lumbar spine MRI, 103 were considered as appropriate, amongst which 68 (66%) scenarios were agreed upon. We prepared a table including some examples of appropriateness of the scenarios which are shown in (Table 3).

**Table 1:** Agreement on Appropriateness of the Scenarios for Lumbar Spine MRI According to the 16 Main Indications: First Round of Consensus Development

	**MRI Indications**	**Total Number of Scenarios**	**Appropriateness of Scenarios**		
			Inappropriate, No. (%)	Uncertain, No. (%)	Appropriate, No. (%)
1	Acute low back pain (onset within the past 6 weeks), spinal infection	16	0	3 (18.75)	13 (81)
2	Acute low back pain ( onset within the past 6 weeks), progressive (objective) neurological signs	2	0 (0)	1 (50)	1 (50)
3	Acute low back pain (onset within the past 6 weeks), suspicion of cauda equina syndrome	4	0 (0)	2 (50)	2 (50)
4	Acute low back pain,( onset within past 6 weeks), suspicion of cancer	6	0 (0)	2 (34)	4 (66.6)
5	Sub-acute low back pain > 6 weeks, at least 6 weeks medical/conservative treatment and spinal infection:	16	0 (0)	6 (38)	10 (62.5)
6	Subacute low back pain > 6 weeks, at least 6 weeks medical/conservative treatment and progressive (objective) neurological signs:	1	0 (0)	0 (0)	1 (100)
7	Sub-acute low back pain > 6 weeks, at least 6 weeks medical/conservative treatment and suspected cauda equina syndrome	2	0 (0)	0 (0)	2 (100)
8	Sub-acute low back pain > 6 weeks, at least 6 weeks medical/conservative treatment and suspicion of cancer	3	0 (0)	3 (100)	0 (0)
9	Sub-acute low back pain > 6 weeks, at least 6 weeks medical/conservative treatment and suspected radiculopathy	3	0 (0)	0 (0)	3 (100)
10	Chronic low back pain (> 3 months), spinal infection	18	0 (0)	0 (0)	18 (100)
11	Chronic low back pain (> 3 months), progressive (objective) neurological signs	3	0 (0)	1 (33.3)	2 (66.6)
12	Chronic low back pain (> 3 months), suspected cauda equina syndrome	6	2 (33.3)	2 (33.3)	2 (33.3)
13	Chronic low back pain (> 3 months), suspicion of cancer	9	1 (11.11)	6 (66)	2 (22.2)
14	Chronic low back pain (> 3 months), suspected radiculopathy	3	0 (0)	0 (0)	3 (100)
15	Chronic low back pain (> 3 months)	2	0 (0)	0 (0)	2 (100)
16	Chronic low back pain (> 3 months), considered a candidate for spine surgery	3	0 (0)	1 (33.3)	2 (66.6)
Total		97	3 (3)	27 (28)	67 (69)

**Table 2:** Agreement on Appropriateness of the Scenarios for Lumbar Spine MRI According to the 30 Main Indications: Second Round of Consensus Development

	**MRI Indications**	**Total Number of Scenarios**	**Appropriateness of Scenarios**		
			Inappropriate, No. (%)	Appropriate, No. (%)	Equivocal, No. (%)
1	Acute low back pain (onset within past 6 weeks), spinal infection	16	0	0	16 (100)
2	Acute low back pain,( onset within past 6 weeks),Progressive (objective) neurological signs	2	0 (0)	1 (50)	1 (50)
3	Acute low back pain,( onset within past 6 weeks), suspected cauda equina syndrome	4	0 (0)	0 (0)	4 (100)
4	Acute low back pain, (onset within past 6 weeks), suspicion of cancer	6	0 (0)	0 (0)	6 (100)
5	Subacute low back pain > 6 weeks, at least 6 weeks medical/conservative treatment and spinal infection:	16	0 (0)	0 (0)	16 (100)
6	Subacute low back pain > 6 weeks, at least 6 weeks medical/conservative treatment and progressive (objective) neurological signs:	1	0 (0)	0 (0)	1 (100)
7	Subacute low back pain > 6 weeks, at least 6 weeks medical/conservative treatment and suspected cauda equina syndrome:	2	0 (0)	0 (0)	2 (100)
8	Subacute low back pain > 6 weeks, at least 6 weeks medical/conservative treatment and suspicion of cancer	3	0 (0)	0 (0)	3 (100)
9	Subacute low back pain > 6 weeks, at least 6 weeks medical/conservative treatment and suspected radiculopathy	3	0 (0)	0 (0)	3 (100)
10	Chronic low back pain (> 3 months), spinal infection	18	0 (0)	0 (0)	18 (100)
11	Chronic low back pain (> 3 months), progressive (objective) neurological signs	3	0 (0)	1 (33.3)	2 (66.6)
12	Chronic low back pain (> 3 months), suspected cauda equina syndrome	6	2 (33.3)	2 (33.3)	2 (33.3)
13	Chronic low back pain (> 3 months), suspicion of cancer	9	1 (11.1)	2 (22.2)	6 (66.6)
14	Chronic low back pain (> 3 months), suspected radiculopathy	3	0 (0)	0 (0)	3 (100)
15	Chronic low back pain (> 3 months)	2	0 (0)	0 (0)	2 (100)
16	Chronic low back pain (> 3 months), the patient is considered a candidate for spine surgery:	3	0 (0)	1 (33.3)	2 (66.6)
17	Traumatic acute disc herniations	1	0 (0)	1 (100)	0 (0)
18	Evaluation posterior ligamentous complexity of spine in type B (distruction) spine injuries	1	0 (0)	1 (100)	0 (0)
19	SCI a, normal X ray, normal CT a and neurological deficit need to MRI a evaluation of spinal cord.	1	0 (0)	1 (100)	0 (0)
20	Postoperative evaluation of failed back syndrome (postoperation residual symptoms).	1	0 (0)	1 (100)	0 (0)
21	Unhealed osteoprotic vertebral compression fracture (MRI without contrast is indicated)	1	0 (0)	1 (100)	0 (0)
22	Kummel's disease of vertebra (MRI without contrast)	1	0 (0)	1 (100)	0 (0)
23	Spinal instability secondary to ligament laxity or torn ligament	1	0 (0)	1 (100)	0 (0)
24	Central cord syndrome (trauma- tumor) syringomyelia	1	0 (0)	1 (100)	0 (0)
25	Traumatic cord injuries	1	0 (0)	1 (100)	0 (0)
26	Myelo neuropathy (vit B12 deficiency, HTLV, neurosyphilis)	1	0 (0)	1 (100)	0 (0)
27	Discitis (MRI with contrast)	1	0 (0)	1 (100)	0 (0)
28	Spinal cord infarction	1	0 (0)	1 (100)	0 (0)
29	Traumatic spine injury with LBP spine MRI without contrast	1	0 (0)	1 (100)	0 (0)
30	Spine surgery with LBP & discopathy & radiculopathy: rule out recurrent discopathy: MRI without and with contrast.	1	0 (0)	1 (100)	0 (0)
Total		111	3 (2.7)	41 (36.9)	67 (60)

^a^Abbreviations: CT, computed tomography; MRI, magnetic resonance imaging; SCI, spinal cord injury

**Table 3:** Examples of the Lumbar Spine MRI Scenario Under Each Indication that Extracted to Identify Appropriateness a by the Expert Panel

A	Acute low back pain (onset within past 6 weeks)
	Spinal infection
	1-having fever, needed to MRI without contrast. A+
	2- Suspicion of systemic or spinal infection, needed to MRI without contrast A?
B	Acute low back pain ( onset within past 6 weeks),
	Progressive (objective) neurological signs
	1-Progressive motor weakness present, needed to MRI without contrast A+
	2- Progressive motor weakness present, needed to MRI with contrast U?
C	Acute low back pain,( onset within past 6 weeks)
	Suspected cauda equina syndrome
	1- Bilateral neurologic signs and symptoms, needed to MRI without contrast: A+
	2- Bilateral neurologic signs and symptoms, needed to MRI with contrast: A?
D	Acute low back pain (onset within past 6 weeks)
	Suspicion of cancer
	1- Unexplained weight loss & failure to improve after one month, needed to MRI without contrast: A+
	2- Unexplained weight loss & failure to improve after one month, needed to MRI with contrast: A+
E	Sub-acute Low back pain > 6 weeks, at least 6 weeks medical/conservative treatment
	Spinal infection
	1- having fever, needed to MRI without contrast: A+
	2- having fever, needed to MRI with contrast: A+
F	Sub-acute low back pain > 6 weeks, At least 6 weeks medical/conservative treatment
	Progressive (objective) neurological signs
	1- Progressive motor weakness present, needed to MRI without contrast: A+
G	Sub-acute low back pain > 6 weeks, At least 6 weeks medical/conservative treatment and
	Suspected cauda equina syndrome
	1-Bilateral neurologic signs and symptoms, needed to MRI without contrast: A+
	2- Acute bladder or bowel dysfunction, needed to MRI without contrast: A+
H	Sub-acute low back pain > 6 weeks, at least 6 weeks medical/conservative treatment
	Suspicion of cancer
	1- Unexplained weight loss & failure to improve after one month, needed to MRI without contrast: A?
I	Sub-acute low back pain > 6 weeks,= At least 6 weeks medical/conservative treatment
	Suspected radiculopathy
	1- Leg pain is > than back pain & pain present in nerve root distribution & positive straight leg raising test < 45º OR positive crossed straight leg raising test, MRI without contrast: A+
J	Chronic low back pain (> 3 months)
	Spinal infection
	1-Suspicion of systemic or spinal infection, MRI with & without contrast: A+
	2-IV drug use , MRI with & without contrast: A+
K	Chronic low back pain (> 3 months)
	Progressive (objective) neurological signs
	1- Progressive motor weakness present, MRI without contrast: A+
	2-Progressive motor weakness present, MRI with contrast: U-
L	Chronic low back pain (> 3 months)
	Suspected cauda equina syndrome
	1-Bilateral neurologic signs and symptoms, MRI without contrast: A+
	2- Acute bladder or bowel dysfunction, MRI without contrast: A+
M	Chronic low back pain (> 3 months)
	Suspicion of cancer
	1-Unexplained weight loss & failure to improve after one month, MRI with & without contrast: A+
	2-Failure to improve after one month & age over 50, MRI with & without contrast: A?
N	Chronic low back pain (> 3 months)
	Suspected radiculopathy
	1-Leg pain is > than back pain& pain present in nerve root distribution & positive straight leg raising test < 45º OR positive crossed straight leg raising test, MRI without contrast: A+
O	Chronic low back pain ( > 3 months):
	1-Suspicion of substantial spinal stenosis on another imaging procedure, MRI without contrast: A+
P	Chronic low back pain ( > 3 months)
	Patient is considered a candidate for spine surgery
	1- Progressive changes in objective neurological findings, MRI without contrast: A+
	2- At least 1 year since last lumbar MRI (without objective change in neurological signs): A? MRI without contrast

^a^A = Appropriate, I = Inappropriate, U = Uncertain, A+ = Agreement, A-= Disagreement, A? = uncertain

## 5. Discussion

To the best of our knowledge, this is the first study that has used the RAND Appropriateness Method for lumbar spine MRI indications. In our study, lumbar spine MRI was considered in 67 (58%) of the scenarios as appropriate, 45 (39%) as uncertain, and three (2.6%) as inappropriate. After two rounds of consensus development, there were still 67 scenarios in which no agreement was reached or the results remained equivocal. This may demonstrate the ambiguous nature of decision making on whether the MRI is indicated for a patient. It may partly explain why it has been so difficult to curb or slow the growth in unnecessary services provided such as the lumbar spine MRI rate around the world (3, 4, 12, 13, 14, 15). Our results will help decision makers in identifying appropriate procedures and focusing their efforts on decreasing unnecessary care. In evidence-based medicine, the question arises that what should be done if there is insufficient evidence for a procedure routinely performed in practice (37). In developed countries, evidence-based clinical guidelines have been helpful, but in middle and low income countries there are serious limitations in preparing these materials (38, 39). Moreover, evidence-based clinical guidelines often lack flexibility and may not provide enough details for clinicians when making decisions about individual patients (38, 40). Formal consensus development (including RAM) provides a timely and efficient solution when evidence is insufficient (40). In our study, we overcame this limitation by developing scenarios representative of the patients seen by clinicians in practice. It should be noted that consensus methods are most widely used in procedures that there is no agreement on. Moreover, no studies were found on using these methods in the lumbar spine MRI. Rashidian (40) performed a review of the evidence in order to identify the indicators of the lumbar spine MRI. General similarities may be observed between the results of the study carried out by Roudsari and the present study. However, our method was more effective since each of the indicators was divided into detailed and accurate scenarios. Accurate and detailed scenarios can be useful for physicians in practice. In some studies, questions remain about the validity of recommendations based on such methods (39). Tan et al.(41) have described the complexities and limitations of using RAM. Many of their criticisms equally apply to other consensus development methods. It should be noted that such methods are useful when there are disagreements or variation in practice and reliable evidence is limited. In these circumstances, formal consensus methods are valuable and their use is inevitable. We spent time on familiarizing the panel members with the method and attracting their valued cooperation. Membership of the panel involves open discussions of personal views and practices and that certain practices might not be supported by evidence or by other panel members. As an advantage of our study, we used the AGREE tool for selection of the evidence sources. This provided a chance for the panel to reach a shared understanding of the evidence before embarking towards consensus building. We also selected the members from different backgrounds and settings to improve comprehensiveness of the views (42, 43). RAM usually results in a long list of scenarios. To ease the use of its results, it may be possible to develop user-friendly software, or to categorize the scenarios into indications and packages in the format of clinical guidelines. We demonstrated that the RAM is useful for development of scenarios for appropriateness of lumbar spine MRI in a developing country. The method should be used more widely in such settings in other areas of healthcare where controversies exist or the practice varies. It also has the added value of developing a level of ownership by the providers if they see their peers and relevant stakeholders are adequately represented in the process.

The findings of this study can be used for developing national guidelines, conducting research to assess whether the criteria are followed in practice and whether their application can curb the growing rate of unnecessary care in all countries. In Iran, a limited amount of resources are allocated to the health sector. Since MRI is an expensive method of medical diagnosis, doctors have to prescribe it after the easier ways of diagnosis are not useful.
